# Effects of physical activity and resilience on anxiety in China children: a mediating model with moderating effects

**DOI:** 10.3389/fspor.2026.1764396

**Published:** 2026-05-26

**Authors:** Yijie Shi, Zibin Wang, Wei Zhao, Mariusz Lipowski, Li Zhuang

**Affiliations:** 1 Gdansk University of Physical Education and Sport, Gdańsk, Poland; 2 Northwest Minzu University, Lanzhou, China; 3 Northwest Normal University, Lanzhou, China; 4 WSB Merito University in Gdańsk, Gdańsk, Poland; 5 Guangdong Vocational College of Science and Technology, Zhuhai, China

**Keywords:** anxiety, Chinese adolescents, parenting styles, physical activity, psychological resilience

## Abstract

**Background:**

Anxiety disorders in children exist on a continuum, ranging from mild symptoms to full clinical diagnoses, underscoring the need for early intervention to reduce long-term effects. Physical activity (PA) has gained attention as a potential, cost-effective intervention. However, the role of psychological resilience (PR) as an intermediary and the impact of parenting styles (PS) as moderators require deeper exploration.

**Objective:**

The aim of this study was to construct a moderated mediation model to explore how physical activity (PA) influences anxiety via perceived resilience (PR), while examining the moderating effects of parenting styles (rejection, emotional warmth, overprotection) in this relationship.

**Participants:**

This study involved a cross-sectional survey of 1,094 Chinese children aged 10–16 years, enrolled in public schools in Zhuhai, Guangdong.

**Setting:**

The participants were selected through a multi-stage stratified cluster random sampling technique to ensure demographic diversity. The sample consisted of 49% male participants, with 43% from primary schools, 43% from junior high schools, and 14% from senior high schools.

**Methods:**

The data were analyzed using SPSS and AMOS software. The PROCESS macro model was employed to investigate the mediating and moderating effects of parental rearing styles, including rejection, emotional warmth, and overprotection.

**Result:**

Mediation and moderated mediation analyses revealed that PA significantly predicted reduced anxiety symptoms. Parenting styles moderated the PA-resilience pathway: (1) emotional warmth strengthened the association (2) rejection and overprotection attenuated it.

**Conclusions:**

Physical exercise alleviates children's anxiety by bolstering mental resilience, with parental rearing styles playing a crucial regulatory role.

## 1 Introduction

Anxiety disorders in children and adolescents are mainly characterized by persistent excessive worry, social avoidance and somatization and other core symptoms. Epidemiological studies indicate that the clinical prevalence of anxiety disorders meeting DSM-5 criteria is approximately 7%–9% (1, 2). Studies have demonstrated a continuum between anxiety symptoms and anxiety disorders: approximately 50% of adult anxiety patients retrospectively report that their core symptoms manifest subclinical features before the age of 11 (3). This continuum from symptom to disorder amplifies the public health significance of early intervention: even anxiety symptoms that do not reach clinical diagnostic thresholds can significantly impair children's learning efficiency (4), peer relationships (5), and subjective well-being (6). Therefore, identifying modifiable protective factors and blocking them at the symptomatic stage through low-cost universal strategies has become a critical pathway to prevent anxiety transformation and reduce disease burden (7, 8).

Physical activity (PA) showed subtype specific benefits in intervention for childhood anxiety. In the case of separation anxiety disorder (SAD), team exercise (e.g., football) relieves symptoms through a dual pathway: situational exposure and autonomy development-children naturally learn independent coping skills in short-term unsupervised exercise scenarios. Randomized controlled trials have shown that group exercise reduces separation anxiety symptoms in children aged 6–9 by 42% (*d* = 0.71) (9); second, sense of control transfer, regular PA improves self-efficacy by strengthening motor skills (such as swimming), cohort studies have confirmed that high-frequency PA (≥5 times/week) can reduce the risk of SAD by 58% (10). These findings highlight the potential of PA as a scalable intervention tool-reshaping children's adaptive responses to anxiety-inducing situations through exercise scenarios, providing a theoretical anchor for developing low-cost prevention programs.

The concept of resilience emerged in developmental psychology during the 1970s, focusing on children's adaptive responses to adversity. Researchers like Norman Garmezy and Emmy Werner discovered that some children continue to show positive developmental outcomes despite growing up in high-risk environments such as poverty and dysfunctional families. This capacity to “thrive in adversity” has been considered the essence of psychological resilience (11). Resilience refers to the ability to effectively cope with stress, trauma, or challenging situations, preventing the onset of stress-related mental health issues such as depression, post-traumatic stress disorder, and anxiety (12, 13). Those with high resilience typically display greater cognitive flexibility and emotional regulation, allowing them to manage negative emotions proactively (14). Conversely, individuals with lower resilience may be more sensitive in social relationships, often impacted by experiences like peer rejection, and tend to engage in social avoidance behaviors. Studies indicate that individuals with social anxiety disorder tend to have lower levels of resilience compared to healthy individuals (15). The positive effect of physical activity on mental resilience is widely supported by research (16, 17). Furthermore, resilience has been shown to mediate the relationship between physical activity and mental health outcomes, including depression and psychological distress (18, 19).

Family environment is the core ecosystem of individual development, and its core elements, parental rearing styles (such as emotional warmth, rejection and overprotection), may indirectly shape the effect boundary of child anxiety intervention by modulating PA's resilience gain effect (20). Research shows that authoritative parenting can enhance PA's promotion of psychological resilience; conversely, authoritarian parenting may weaken PA's adaptive benefit through threat perception enhancement (21). In addition, overprotective parenting may hinder the development path of self-efficacy in PA situations (such as replacing children to solve sports challenges) by limiting children's opportunities for autonomous exploration, but its interaction with PA is unclear (22). It is worth noting that existing studies have examined the independent effects of PA or parenting styles on resilience (e.g., PA enhances resilience through neuroplasticity (23); emotional warmth enhances resilience through secure attachment (24), but there is still no empirical test on how the two synergize in the chain path of PA → resilience → anxiety. This theoretical fragmentation leads to gaps in family-exercise intervention design: failure to identify which parenting patterns maximize PA's anxiety-reducing benefits.

**Figure 1:**
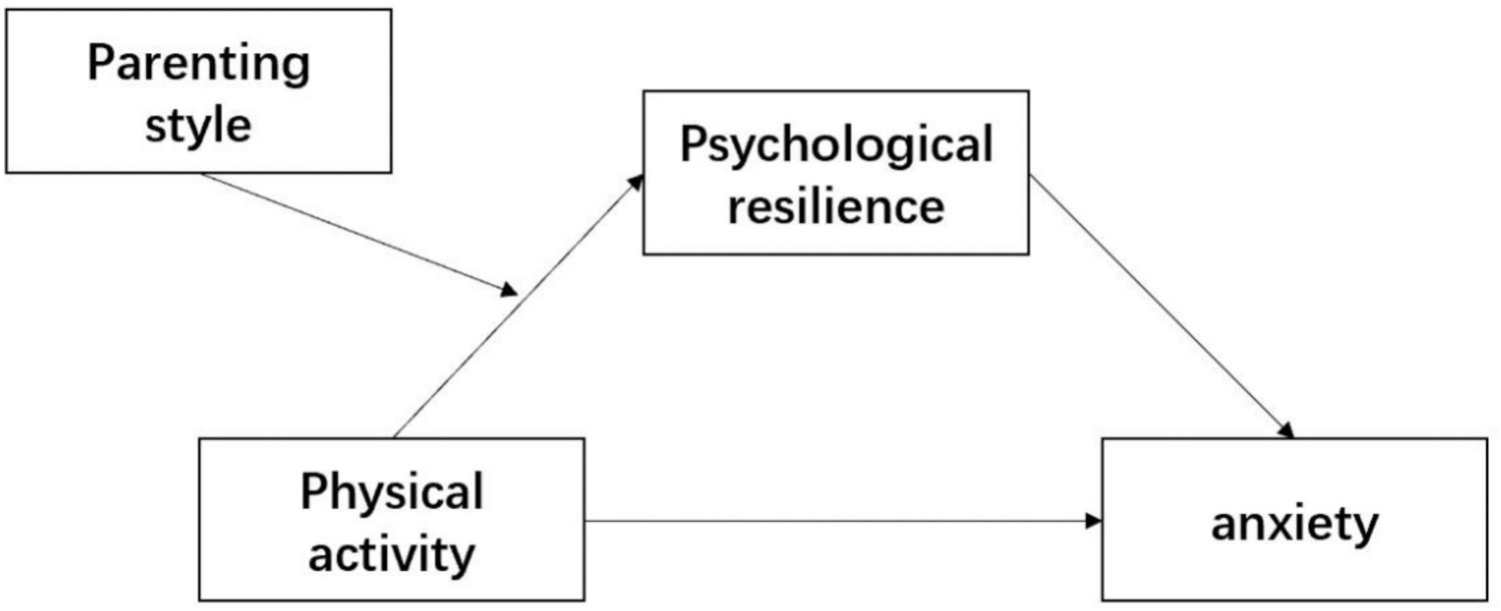
The proposed moderated mediation model to be tested.

This study aims to develop a moderated mediation model to explore the role of parental rearing styles in influencing the pathway from physical activity (PA) to resilience and anxiety. The goal is to establish a mechanism for creating contextually appropriate exercise intervention strategies. Specifically, the study seeks to examine how parental rearing styles regulate the relationship between PA and anxiety through resilience, providing support for family-school collaborative interventions. In this context, the study investigates the connections between adolescent physical health, parenting styles, resilience, and anxiety. Drawing on prior research, we propose three hypotheses: (1) physical health is inversely related to anxiety in children; (2) resilience acts as a mediator between physical activity and anxiety; and (3) parenting styles influence the indirect effect of physical activity on anxiety through resilience. Based on these hypotheses, a theoretical model is proposed for testing (Figure 1), where physical activity improves mental resilience and reduces anxiety. The relationship between PA and anxiety is mediated by resilience, and the strength of this mediation is contingent on the influence of parental rearing styles.

## 2 Research technique

### 2.1 Participants

This study is a cross-sectional observational survey; therefore, all reported effects represent statistical associations and do not establish causality. Inclusion criteria: age 10–16 years old, full-time school students (school registration system verification), no serious physical diseases (such as congenital heart disease, polio, etc.) or mental diseases.

Exclusion criteria: 1) physical and mental health abnormalities: patients with congenital or acquired severe physical diseases (such as congenital heart disease, nervous system diseases), history of mental diseases (such as depression, schizophrenia) or acute infection period; 2) data quality defects: participants with insufficient cooperation degree (such as incomplete scale answers, incomplete experimental tasks) or voluntary withdrawal, resulting in invalid data.

**Figure 2:**
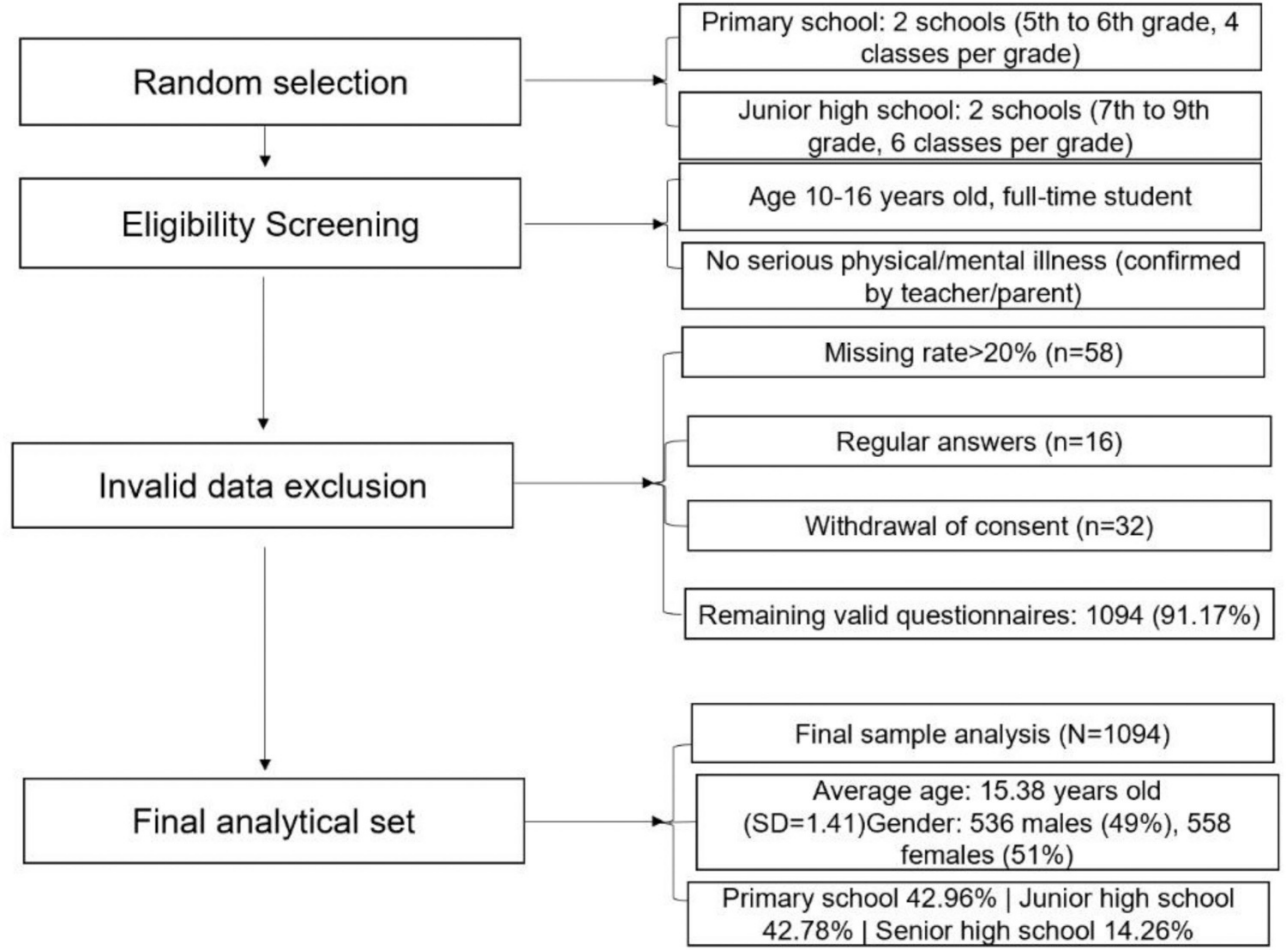
Flowchart of subject screening.

This study received approval from the Ethics Committee of [School of Teacher Education, Northwest Minzu University] (approval No. [NWNU-EDU-IRB-2023-0316). Written informed consent was obtained from the legal guardians of all participants. Additionally, written assent was provided by all child and adolescent participants after they were given age-appropriate information regarding the study procedures and their rights, including the right to withdraw at any time without consequence. A multi-stage stratified cluster random sampling technique was employed in this study. The public schools in Zhuhai City, Guangdong Province (including key schools and ordinary schools) were selected randomly. Two primary schools (grade 5 to grade 6, 4 classes per grade) and two junior high schools (grade 7 to grade 9, 6 classes per grade) were randomly selected, and 48 classes (16 classes in primary schools and 36 classes in junior high schools) were included. All the students in the class who were 10–16 years old, full-time in school and without serious physical or mental illness (confirmed by teachers/parents) were invited. A total of 1,200 questionnaires were distributed. After recovery, invalid data with missing rate >20% (*n* = 58), regular answer (such as 10 consecutive questions with the same option, *n* = 16) and withdrawal of consent (*n* = 32) were excluded. Finally, 1,094 questionnaires were included in the analysis (effective rate 91.17%). Missing values were handled by censoring (missing <5%) and no data imputation was performed. The final sample (*N* = 1,094) had a mean age of 15.38 years (SD = 1.41), 536 boys (49%) and 558 girls (51%), covering grades 5 to 1 of senior high school (42.96% of primary, 42.78% of junior high, and 14.26% of senior high). The participant recruitment and screening process is illustrated in Figure 2.

### 2.2 Measuring tool

#### 2.2.1 Measurement of physical activity

The Chinese version of the Physical Activity Rating Scale (PARS-3) was utilized, consisting of three assessment criteria: exercise intensity, duration of a single session, and frequency of weekly exercise. The scale employs a three-point scoring system: “1 = light or short duration,” “2 = moderate,” and “3 = high intensity or long duration.” The total score ranges from 0 to 100, with higher scores indicating more physical activity. The Chinese PARS-3 has demonstrated strong reliability and validity in measuring exercise load (44). In this study, the Cronbach's alpha coefficient for the Physical Activity Scale was 0.774.

#### 2.2.2 Measurement of psychological anxiety

In this study, the Chinese version of the Social Anxiety Scale for Children (SASC) was used, consisting of 10 items that measure two main aspects: fear of negative evaluation and social avoidance/distress. Participants' responses are rated on a three-point scale: “0 = never,” “1 = sometimes,” and “2 = always.” The total score ranges from 0 to 20, with higher scores reflecting a greater tendency toward social anxiety. The Chinese version of the SASC has been validated for use in children, showing good reliability and validity (45). In this study, the Cronbach's alpha for the anxiety scale was 0.806.

#### 2.2.3 Measurement of mental resilience

This study utilized the Chinese version of the Connor-Davidson Resilience Scale (CD-RISC) (46), which includes 25 items designed to assess three aspects of resilience: tenacity, strength, and optimism. The scale employs a five-point Likert system with the following response options: “0 = completely incorrect,” “1 = rarely correct,” “2 = sometimes correct,” “3 = often correct,” and “4 = almost always correct” (46). The total score ranges from 0 to 100, with higher scores indicating greater resilience. The Chinese adaptation of the CD-RISC has been extensively validated for adolescent populations in China, showing excellent reliability and validity (47, 48). In the present study, the Cronbach's alpha coefficient was 0.946. Confirmatory factor analysis (CFA) revealed a good model fit (see Table 1), with the following indices: *X*^2^/*df* = 1.144, RMSEA = 0.011, CFI = 0.998, TLI = 0.997, and ITL = 0.998.

**Table 1:** Resilience fit test.

Index	Reference standard	Results
CMIN/DF	1–3 for outstanding, 3–5 for good	1.144
RMSEA	<0.05 for outstanding, <0.08 for good	0.011
ITL	>0.9 for outstanding, >0.8 for good	0.998
TLI	>0.9 for outstanding, >0.8 for good	0.997
CFI	>0.9 for outstanding, >0.8 for good	0.998

#### 2.2.4 Measurement of parenting style

Parenting styles were assessed using the short form of the Egna Minnen Barndoms Uppfostrand (S-EMBU) scale (49), which includes 23 items measuring three dimensions: rejection, overprotection, and emotional warmth. Participants rated their responses on a four-point scale: “1 = never,” “2 = occasionally,” “3 = often,” and “4 = very often.” Higher scores in each dimension reflect more frequent application of the respective parenting style. The Chinese version of the S-EMBU has been thoroughly validated for adolescent populations in China, showing satisfactory reliability and validity. In this study, the Cronbach's alpha coefficient for the Parental Rearing Style Scale was 0.771. Confirmatory factor analysis (CFA) demonstrated a good fit for the three-factor model (Table 2), with the following indices: *X*^2^/*df* = 1.152, RMSEA = 0.012, CFI = 0.998, TLI = 0.997, and ITL = 0.998.

**Table 2:** Parental style fit test.

Index	Reference standards	Results
CMIN/DF	1–3 for outstanding, 3–5 for good	1.152
RMSEA	<0.05 for outstanding, <0.08 for good	0.012
ITL	>0.9 for outstanding, >0.8 for good	0.998
TLI	>0.9 for outstanding, >0.8 for good	0.997
CFI	>0.9 for outstanding, >0.8 for good	0.998

#### 2.2.5 Statistical analysis

Data analysis was performed using IBM SPSS 27.0 and AMOS software (25). Descriptive statistics and appropriate statistical tests were applied according to the nature of the variables. For normally distributed data, the results are expressed as mean ± SD, and group differences were examined using an independent samples *t*-test. For skewed data, results are presented as median (interquartile range) [M (P25, P75)], with group comparisons conducted using the Mann–Whitney *U* test. Categorical variables are reported as frequency and percentage [*n* (%)], and group differences were analyzed using the *χ*^2^ test. The relationship between variables was assessed using Pearson's correlation coefficient, with statistical significance determined at *α* = 0.05.

The primary analysis focused on evaluating the mediating role of resilience and the moderating effect of parenting styles. Initially, a simple mediation model was tested using Hayes' PROCESS macro (Model 4). After controlling for variables such as age, sex, and other sociodemographic factors, physical activity (PA) was designated as the predictor, anxiety as the outcome, and psychological resilience (PR) as the mediator. The significance of the mediation effect was determined using the bootstrap method with 5,000 resamples. The findings revealed that PA reduced anxiety symptoms indirectly by increasing levels of PR [indirect effect *β* = −0.18, 95% CI (−0.25, −0.11)].

The moderating effect of parental rearing style (PS) was further analyzed by Model7. The moderating effects of three dimensions of PS scale, namely, Reject, EW and OP, on PA → PR pathway were examined. In order to avoid interpretation bias caused by reverse scoring items, the item orientation of S-EMBU scale was uniformly corrected before data analysis. The moderating effect test showed that EW significantly enhanced PA's facilitation of PR (interaction *β* = 0.12, *p* = 0.032), while overprotection weakened the path effect (*β* = −0.09, *p* = 0.046), suggesting that parenting styles had situation-specific moderating function in exercise intervention.

## 3 Result

### 3.1 Sociodemographic characteristics of the participants

**Figure 3:**
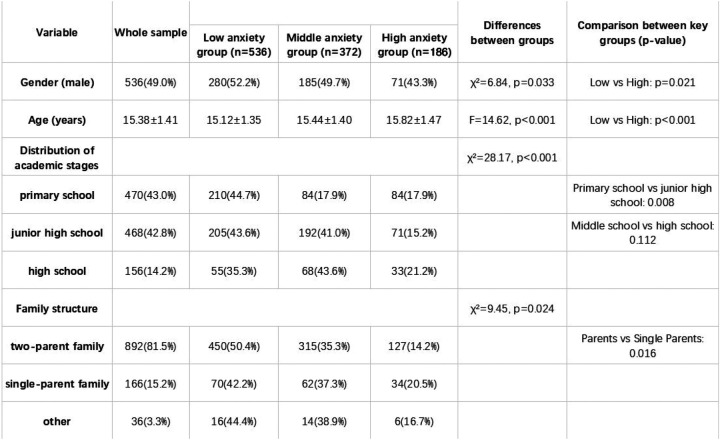
Basic information about participants.

The sociodemographic characteristics of the participants are presented in Figure 3.

### 3.2 Correlation between study variables

**Figure 4:**
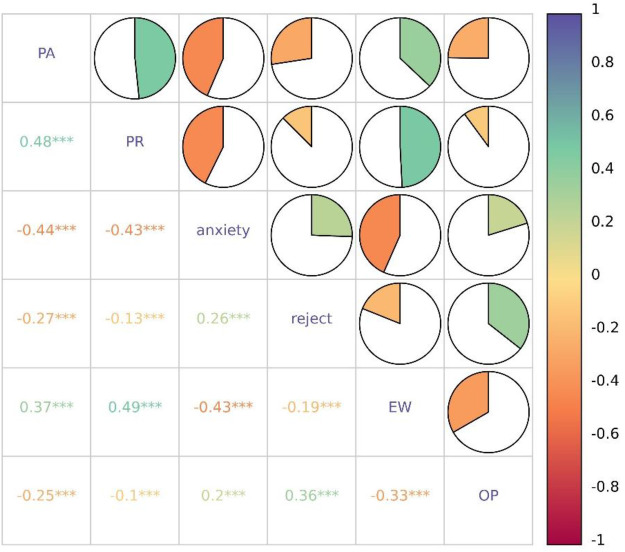
Descriptive statistics, correlation analysis results.

In this analysis, an exploratory analysis was performed by Pearson correlation analysis of correlations among multiple variables. [The S-EMBU scale includes reverse scoring, and analyzes the three dimensions of Reject, Emotional Warmth (EW) and Overprotection (OP) one by one.] PA was positively correlated with PR and anxiety, and negatively correlated with PS. PS reject and OP were negatively correlated with PR, EW was positively correlated with PR. The descriptive statistics and correlation coefficients among the study variables are shown in Figure 4.

### 3.3 Mediation analysis (*B*, 95% CI, BETA, SE, *T*, *p*)

The results (see Tables 3, 4) indicate that physical activity (PA) significantly predicts anxiety (*B* = −0.435, *t* = 15.297, *p* < 0.01). When the mediating variable is considered, PA continues to have a significant direct effect on anxiety (*B* = −0.299, *t* = 6.33, *p* < 0.01). PA also positively predicted psychological resilience (PR) (*B* = 0.35, *t* = 18.25, *p* < 0.01), while PR negatively predicted anxiety (*B* = 0.48, *t* = −9.43, *p* < 0.01). Moreover, the 95% bootstrap confidence intervals for both the direct effect of PA on anxiety and the mediating effect of PR did not include zero (see Tables 3, 4), suggesting that PA not only directly influences anxiety but also affects anxiety through PR. The direct effect (0.30) and the indirect effect (0.14) accounted for 68.61% and 31.39% of the total effect (0.44), respectively.

**Table 3:** Path coefficient test of psychological resilience model.

Outcome	Predictive variables	R-sq	*F*	*β*	*T*
PR	PA	0.2338	333.211**	0.483	18.254**
Anxiety	PA	0.2505	182.365**	−0.299	−9.972
	PR			−0.283	−9.434
Anxiety	PA	0.1894	255.151**	−0.435	15.973**

**Table 4:** Bootstarp mediating effect test results.

Effect relationship	Effect value	SE	LLCI	ULCI	Relative effect value
Total effect	−0.435	0.021	−0.489	−0.382	
Direct effect	−0.299	0.017	−0.357	−0.240	68.61%
Mediation effect	−0.137	0.016	−0.173	−0.101	31.39%

### 3.4 Moderator analysis

The results indicated that parental rearing styles, particularly rejection and overprotection, diminished the positive impact of physical activity on mental resilience, while emotional warmth had a strengthening effect. Specifically, the positive influence of physical activity on mental resilience was reduced when parental rejection was high (*B* = −0.09, *t* = −4.01, *p* < 0.01). On the other hand, when parents exhibited higher levels of emotional warmth, the positive effect of physical activity on mental resilience was enhanced (*B* = 0.08, *t* = 2.40, *p* < 0.05). Additionally, increased overprotection by parents also weakened the positive predictive effect of physical activity on mental resilience (*B* = −0.09, *t* = −4.01, *p* < 0.01). These results highlight the significant role of parenting styles in shaping adolescent resilience, suggesting that efforts to foster resilience should focus on reducing rejection and overprotection while promoting emotional warmth.

The results of the moderating effect analysis, as shown in Table 5, reveal that the moderating effect of parental rejection causes physical activity (PA) to have a stronger positive influence on psychological resilience (PR) when parental rejection is low (M − 1SD). Simple slope analysis indicates an effect value of 0.549 (*t* = 12.37, *p* < 0.001). However, when parental rejection is high (M + 1SD), PA's predictive effect on PR significantly decreases to 0.363 (*t* = 8.15, *p* < 0.001), representing a 33.7% reduction. This suggests that increased parental rejection weakens the impact of PA on resilience (*β* = −0.092, *p* < 0.001). In the analysis of affective warmth, PA predicted PR at a level of 0.537 (*t* = 11.89, *p* < 0.001) under low affective warmth (M−1SD), while the effect decreased to 0.382 (*t* = 7.64, *p* < 0.001) under high warmth (M + 1SD). This implies that affective warmth may partially counteract the effect of PA through an alternative support mechanism (*β* = 0.132, *p* < 0.001). Furthermore, the moderating effect of overprotective parenting showed that PA most strongly promoted PR (*b* = 0.549) when overprotective parenting was low (M − 1SD). However, the effect weakened to 0.363 (*β* = −0.093, *p* = 0.002) at high levels of overprotection (M + 1SD), similar to the effect of parental rejection. The moderating effects of three parenting styles were differentiated: rejection and overprotection significantly inhibited PA → PR pathway (Cohen's *f*^2^ = 0.15), while emotional warmth increased PR's conversion efficiency to PA, which made the model explanatory power (*R*^2^ = 0.357) reach clinical significance threshold. When parental rejection was 1 SD higher than the mean, an additional 30% PA intervention was needed to compensate for the inhibitory effect.

**Table 5:** Mediated model test with regulation.

Outcome	Predictive variables	*β*	*T*	R-sq	*F*
PR				0.239	113.791***
	PA	0.459	15.649***		
	anxiety	−0.003	−0.132		
	PA*anxiety	−0.077	−2.578		
PR				0.357	201.638**
	PA	0.282	9.092***		
	EW	0.404	14.383***		
	PA*EW	0.132	4.005***		
PR				0.241	115.241***
	PA	0.456	15.602***		
	OP	0.002	0.080***		
	PA*OP	−0.092	−3.084		

The moderating mediating effect of parental rejection was −0.152 (95% CI: −0.195 to −0.112) when parental rejection was low (M − 1SD), and significantly reduced to −0.108 (95% CI: −0.142 to−0.077) when parental rejection was high (M + 1SD), a reduction of 21.9%. The intergroup comparison showed that the mediating effect difference between high and low rejection levels was 0.044 (95% CI: 0.016–0.078), indicating that the mediating path strength of PA → PR decreased by nearly a quarter for each standard deviation of parental rejection. In the moderating analysis of emotional warmth upbringing, the mediating effect was −0.043 (95% CI: −0.085 to −0.001) at low emotional warmth levels (M − 1SD), while the effect was significantly enhanced to −0.117 (95% CI: −0.151 to −0.088) at high emotional warmth levels (M + 1SD), an increase of 72.1%. This two-way adjustment showed that for each SD increase in emotional warmth, the absolute value of the mediating effect increased 1.7-fold (high vs low difference = −0.074, 95% CI: −0.121 to −0.030). In addition, the moderating effect of overprotection was −0.155 (95% CI: −0.197 to −0.115) at low overprotection level (M − 1SD), but weakened to −0.103 (95% CI: −0.140 to −0.070) at high overprotection level (M + 1SD), a decrease of 33.5%. Rejection and overprotection significantly inhibited the PA → PR mediated pathway (difference of effect was 0.044 and 0.053, respectively), while emotional warmth increased the PR mediated pathway efficiency (Δ*R*^2^ = 0.118). When parenting styles were at high levels of rejection or overprotection, an additional 30%–35% PA dose was needed to achieve baseline mediating effect intensity. The conditional indirect effects of parenting styles at different levels (M – 1SD, M, and M + 1SD) are presented in Table 6.

**Figure 5:**
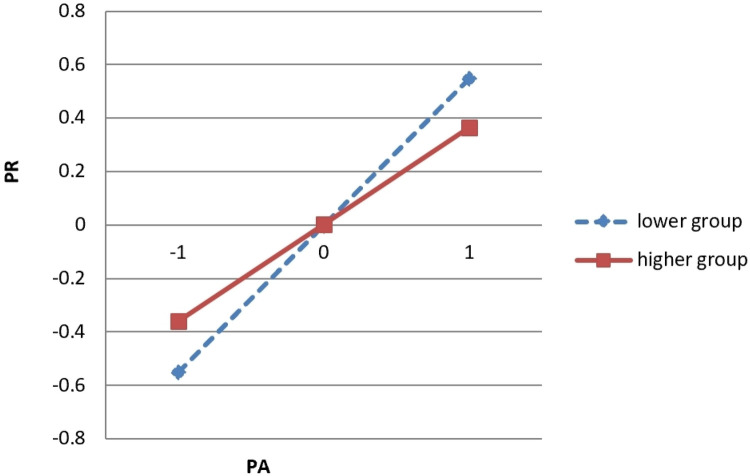
The moderating effect of reject between PA and PR.

**Figure 6:**
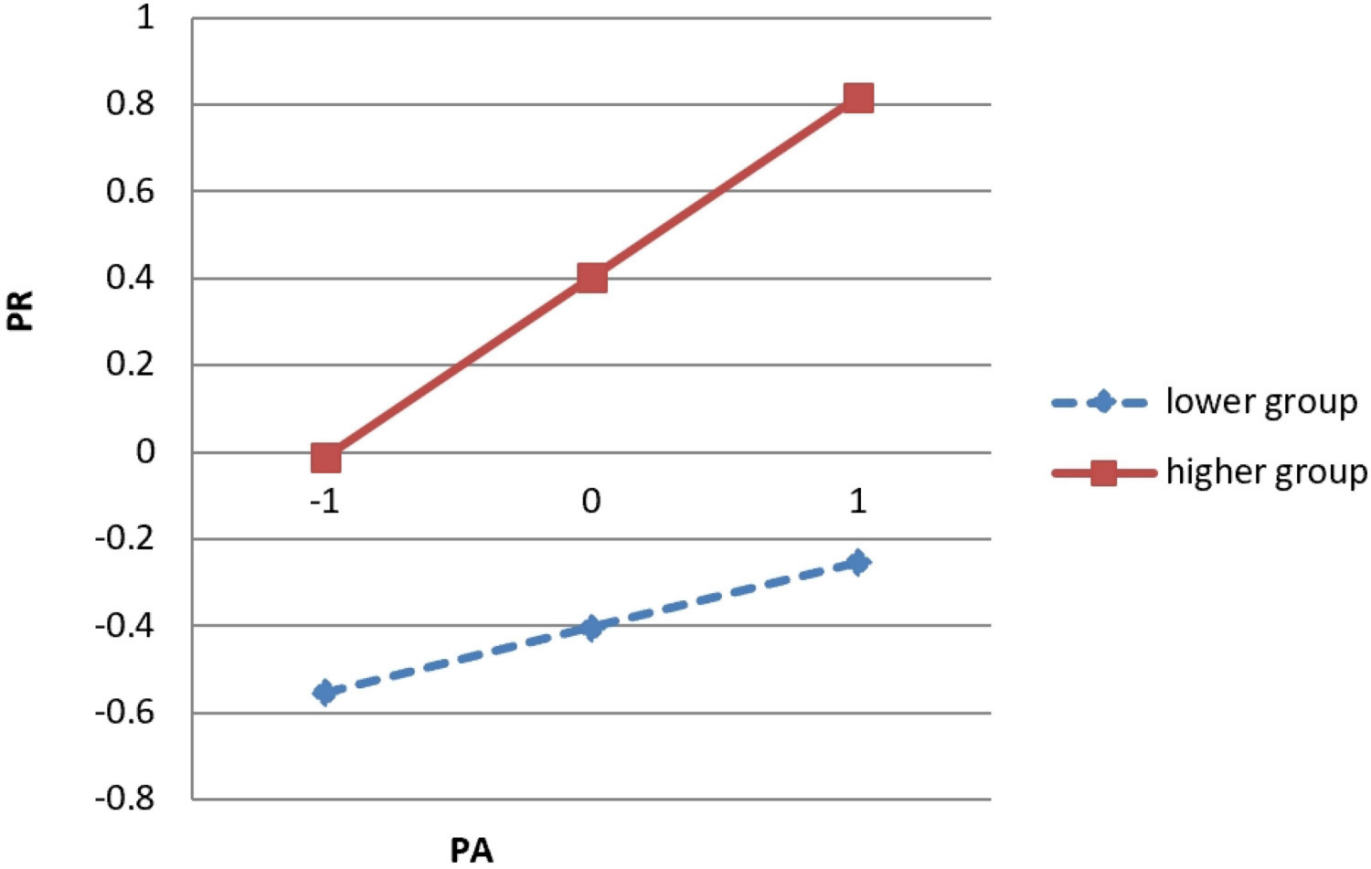
The moderating effect of emotional warmth between PA and PR.

**Figure 7:**
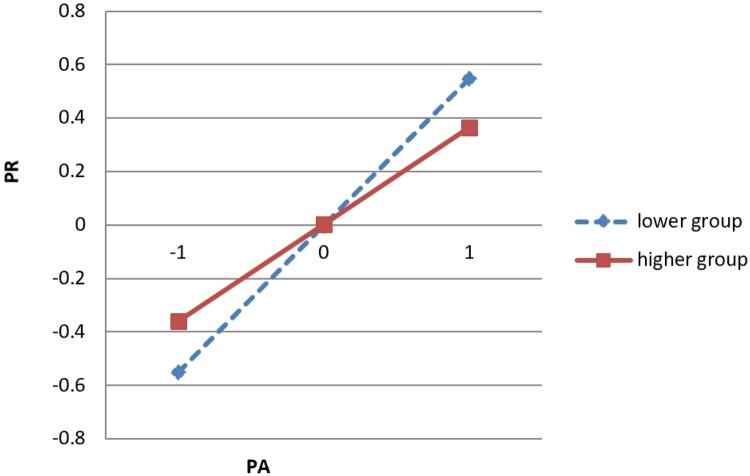
The moderating effect of overprotection between PA and PR.

1. Regression analysis (adjusting variable is reject) showed that PA had significant positive main effect on outcome variable [*b* = 0.459, *p* < 0.001, 95% CI (0.402, 0.517)], indicating that the higher PA level, the higher PR level. The study found that rejection had a significant moderating effect [interaction term *b* = −0.078, *p* = 0.010, 95% CI (−0.137, −0.019)], i.e., the positive effect of PA on PR gradually weakened as the level of rejection increased. Simple slope analysis further revealed that PA had a stronger effect on PR (*b* = 0.537) under low reject conditions (reject = −1) and a significantly lower effect (*b* = 0.382) under high reject conditions (reject = 1). The moderating effect of parental rejection on the relationship between PA and PR is illustrated in Figure 5.
2. Regression analysis (EW as moderator) showed that PA had a significant positive main effect on PR (*b* = 0.459, *p* < 0.001), while EW had a significant moderating effect (*b* = − 0.078, *p* = 0.010). Simple slope analysis showed that PA promoted PR more strongly (*b* = 0.537) under low-affective warmth conditions (*Z* = −1), while its effect weakened (*b* = 0.382) under high-affective warmth conditions (*Z* = 1). The moderating effect of emotional warmth on the relationship between PA and PR is shown in Figure 6.
3. Regression analysis with OP as the adjusting variable showed that PA had a significant positive main effect on PR [*b* = 0.456, *p* < 0.001, 95% confidence interval (0.399, 0.513)]. At the same time, a significant moderating effect of OP was observed [interaction term *b* = −0.093, *p* = 0.002, 95% confidence interval (−0.152, −0.034)], indicating that the positive effect of PA on PR gradually weakened as OP levels increased. Simple slope analysis showed that PA promoted PR more strongly (*b* = 0.549) under low overprotection (Op = −1), while its effect was significantly reduced (*b* = 0.363) under high overprotection (Op = 1) (Figure 7).

**Table 6:** Comparison of moderated mediating effects of parenting styles on different dimensions.

*Z* reject	Different levels/groups	Effect	BootSE	BootLLCI	BootULCI
Mediated effect	eff (M − 1SD)	−0.1516	0.021	−0.1951	−0.1117
	eff2 (M)	−0.1297	0.0173	−0.1649	−0.0964
	eff3 (M + 1SD)	−0.1078	0.0167	−0.1423	−0.0765
Comparison of mediated effects	eff2 − eff1	0.0219	0.0078	0.008	0.0389
	eff3 − eff1	0.0438	0.0156	0.016	0.0778
	eff3 − eff2	0.0219	0.0078	0.008	0.0389
*Z* EW					
Mediated effect	eff (M − 1SD)	−0.0425	0.021	−0.0853	−0.0008
	eff2 (M)	−0.0797	0.0143	−0.1081	−0.0535
	eff3 (M + 1SD)	−0.1169	0.0155	−0.1506	−0.0884
Comparison of mediated effects	eff2 − eff1	−0.0372	0.0116	−0.0605	−0.0151
	eff3 − eff1	−0.0744	0.0232	−0.121	−0.0302
	eff3 − eff2	−0.0372	0.0116	−0.0605	−0.0151
*Z* Op					
Mediated effect	eff (M − 1SD)	−0.155	0.0213	−0.1966	−0.1148
	eff2 (M)	−0.1288	0.0182	−0.1661	−0.0951
	eff3 (M + 1SD)	−0.1025	0.0175	−0.1402	−0.0702
Comparison of mediated effects	eff2 − eff1	0.0263	0.0071	0.0139	0.0416
	eff3 − eff1	0.0525	0.0142	0.0278	0.0832
	eff3 − eff2	0.0263	0.0071	0.0139	0.0416

## 4 Discussion

This study explored the mechanism underlying the relationship between physical activity and anxiety symptoms by developing a mediation model. First, the observed negative correlation between physical activity and anxiety aligns with the Body-Mind Synergy Theory, which posits that physiological factors play a role in mental adaptation (26). Second, the Bootstrap test revealed that resilience served as a significant mediator between physical fitness and anxiety, suggesting that children with higher physical fitness may lower their anxiety risk by improving their ability to cope with stress, such as through emotional regulation and cognitive flexibility (27). Furthermore, parental rearing styles were found to significantly moderate the latter part of this mediating pathway. When children's physical activity met the WHO's recommended level (≥60 min/day), democratic parenting was more effective in helping children translate physical fitness into psychological resilience by offering autonomy-supportive guidance (28). Conversely, authoritarian or permissive parenting styles did not significantly influence this pathway, supporting the “resource-demand matching hypothesis” in family systems theory (10). These findings suggest that addressing anxiety in physically active children requires collaboration between parents and schools, incorporating resilience training into exercise curricula, and encouraging parents to adopt a “supportive supervision” approach to enhance the effectiveness of the mediating mechanism (29).

This study confirms a negative correlation between physical activity levels and anxiety symptoms in children, consistent with most studies (30–32). The underlying mechanism may involve physical activity enhancing cognitive reappraisal of anxiety-induced stimuli by up-regulating the effectiveness of the prefrontal cortex in regulating the amygdala, rather than relying solely on subjective positive cognition. However, the meta-analysis by Rodriguez-Ayllon et al. (33). showed that the overall effect size of physical activity on anxiety was not significant [*g* = 0.01, 95% CI (−0.10, 0.12)], suggesting heterogeneity between studies (34). This may stem from three factors:

First, there were significant differences in the sensitivity of measurement instruments-single dimension scales (e.g., GAD-7) may underestimate the effect of physical activity on specific anxiety subtypes (e.g., separation anxiety), while the Multidimensional Child Anxiety Scale (MCAS) in this study could identify the dimension of motion-related anxiety (e.g., social avoidance and somatic alertness); second, the moderating effect of activity type was ignored, e.g., team sports such as football/basketball through synchronous social interaction (e.g., tactical collaboration, nonverbal emotional communication) activate oxytocin release pathway (35), its antianxiety benefit may be significantly higher than that of individual activities (e.g., running), but the existing review does not subdivide the activity type (34); in addition, developmental stage specific influence results, prepubertal children (10–16 years old) have higher neuroplasticity, and their BDNF level responds to motor intervention stronger than adult population (36). Future randomized controlled trials are needed to test whether long-term planned physical exercise programs reduce anxiety through these neural mechanisms and to further examine the role of cultural environments (such as family common exercise frequency) in regulating the generalization of effects.

Our mediating model suggests that physical activity may indirectly reduce anxiety symptoms in children by increasing resilience. Structured physical activity promotes resilience through a two-channel behavioral-cognitive model, which in turn steps down anxiety symptoms in children (37). This finding echoes longitudinal studies of exercise interventions promoting mental resilience (35, 36, 38), particularly Smith et al. (50) finding an association between exercise dose and cortisol rhythm, further supporting physical activity as a viable pathway for mental health promotion in children. Notably, resilience also exhibits significant mediating effects on the stress-anxiety pathway (39, 40), suggesting that resilience has a two-way regulatory function-enhancing stress resilience by enhancing positive coping resources such as self-efficacy. This dynamic adaptive feature was validated in the subgroup analysis of this study, with a normalized effect value (Cohen's *d* = 0.45) reaching the threshold of clinical significance, suggesting that resilience training should be a central target for anxiety intervention (39). Based on the above evidence chain, we suggest that a dual-mode strategy of “exercise intervention-resilience training” should be adopted: in the early stage, structured exercise courses (such as 60 min of moderate and high intensity activities per day) should be used to improve the physiological regulatory basis, and in the later stage, cognitive reconstruction training should be combined to strengthen psychological adaptive resources, so as to achieve stepwise improvement of anxiety symptoms through multi-system cooperation.

This study analyzed the situational mechanism of the relationship between physical activity and mental resilience through a moderated mediation model, confirming the core claim of family ecosystem theory, that is, parental rearing patterns as proximal environmental factors significantly modulate the psychological benefits of exercise intervention (41). Unlike most current studies focusing on individual-level variables (42), we found that emotional warmth, by buffering HPA axis overreaction (26), enables children with high physical activity to maintain resilience levels in stressful situations. While overprotective parenting weakens the virtuous cycle of motor behavior and self-efficacy (43), especially during preadolescence when autonomy needs are high. These findings are groundbreaking: the effect of physical activity on anxiety is not universal, and peaks when parents provide moderate challenge (30 min of autonomous exercise decisions per day) and high emotional feedback.

In this cross-sectional sample of Chinese children and adolescents, higher physical activity was associated with lower anxiety symptoms, partly through resilience, and this pathway varied across parenting styles. These findings highlight resilience as a potentially important psychosocial pathway and suggest that family context may shape the strength of PA-related benefits. Future research should employ longitudinal and randomized controlled designs, incorporate objective physical activity measures (e.g., accelerometry) and, where feasible, biological indicators of stress regulation, to test temporal ordering and causal mechanisms and to evaluate generalizability across diverse cultural and family environments.

### 4.1 Limitations and future directions

The limitations of this study are as follows: ① Cross-sectional design cannot infer causal relationship, so it is necessary to track the dynamic interaction between PA and anxiety longitudinally; ② Self-reported data may be affected by social approval bias, so objective measures (such as accelerometer and teacher evaluation) should be combined in the future; ③ Samples are only from public schools in China cities, and the results should be cautious when extended to rural or cross-cultural groups.

For practical enlightenment, it is suggested to adopt a family function step-by-step intervention model: first, improve parents “exercise support effectiveness” through parent-child exercise curriculum, then use motivational interview technology to reduce overprotection, and finally establish a positive feedback loop of ‘exercise achievement-resilience enhancement-parenting optimization.’ This is highly consistent with the concept of ‘life course health promotion’ advocated by WHO (51), emphasizing the sustainable transformation of health behaviors through intergenerational interaction.

## Data Availability

The original contributions presented in the study are included in the article/Supplementary Material, further inquiries can be directed to the corresponding author.
